# 5-Phenyl-1,2,5-di­thia­zepane

**DOI:** 10.1107/S1600536814002207

**Published:** 2014-02-12

**Authors:** Lauren A. Mitchell, Michelle L. Mejía, Seyma Gören Keskin, Bradley J. Holliday

**Affiliations:** a Department of Chemistry, The University of Texas at Austin, 105 E 24th Street, Stop A5300, Austin, Texas 78712, USA

## Abstract

In the title compound, C_10_H_13_NS_2_, the seven-membered ring adopts a chair conformation. The S—S bond length is 2.0406 (5) Å and the C—S—S—C torsion angle is −83.89 (7)°. The amine group is *sp* ^2^-hybridized. In the crystal, mol­ecules are linked into chains along [001] by weak inter­molecular S⋯S contacts of 3.5246 (5) Å.

## Related literature   

For properties of di­sulfide compounds, see: Pazderlová *et al.* (2012 ▶). For similar compounds, see: Roze *et al.* (2006 ▶); Bulavin (1971 ▶). For related structures, see: Pickardt *et al.* (2006 ▶); Capasso *et al.* (1977 ▶). For standard bond lengths, see: Allen *et al.* (1987 ▶). For previous reports of S⋯S inter­actions, see: Chen *et al.* (2009 ▶); Reinheimer *et al.* (2009 ▶). For the calculation of the functionality of the amine group in terms of hybridization, see: Allen *et al.* (1995 ▶). For the synthesis, see: Elderfield *et al.* (1958 ▶).
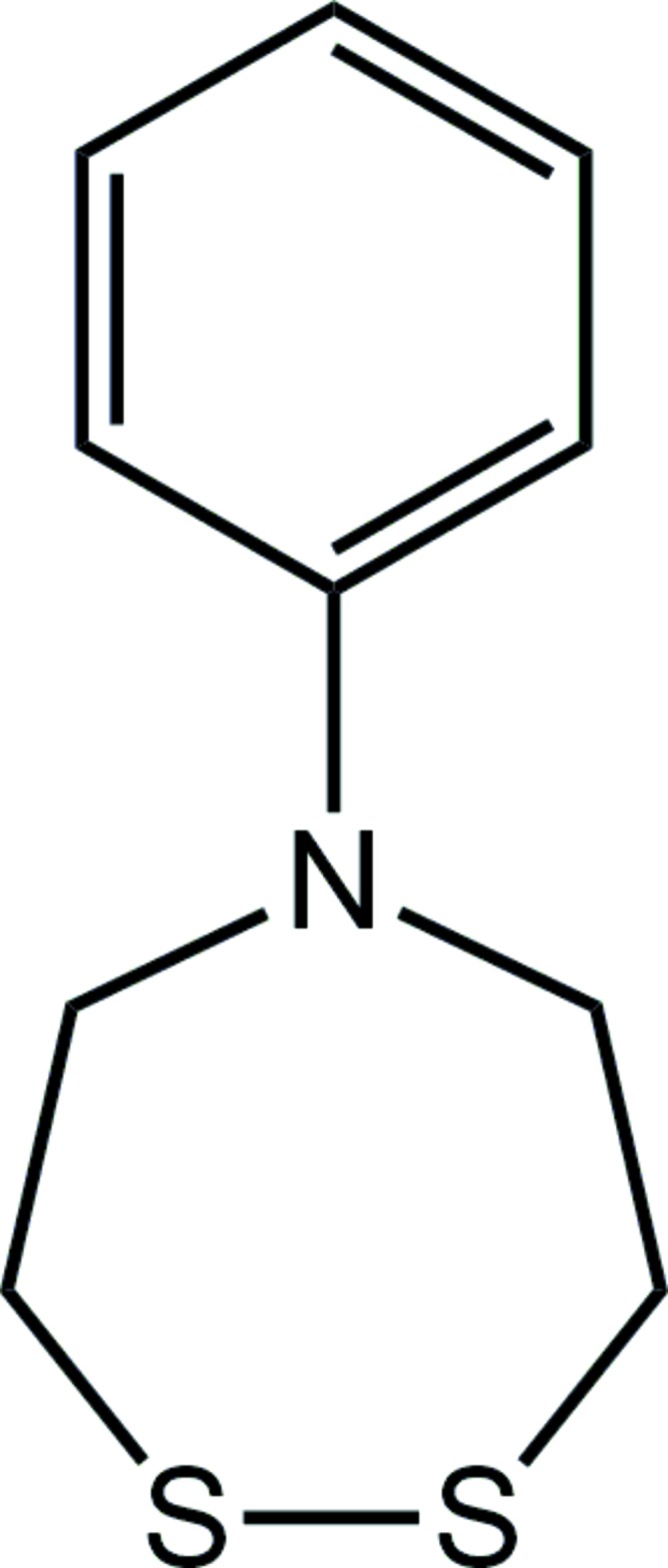


## Experimental   

### 

#### Crystal data  

C_10_H_13_NS_2_
*M* *_r_* = 211.33
Monoclinic, 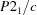

*a* = 9.5760 (2) Å
*b* = 12.2310 (3) Å
*c* = 9.9811 (2) Å
β = 120.392 (2)°
*V* = 1008.38 (4) Å^3^
*Z* = 4
Mo *K*α radiation
μ = 0.48 mm^−1^
*T* = 153 K
0.50 × 0.30 × 0.20 mm

#### Data collection  

Nonius Kappa CCD diffractometer
Absorption correction: multi-scan (*DENZO* and *SCALEPACK*; Otwinowski & Minor, 1997 ▶) *T*_min_ = 0.856, *T*_max_ = 1
3055 measured reflections
1763 independent reflections
1675 reflections with *I* > 2σ(*I*)
*R*_int_ = 0.012

#### Refinement  

*R*[*F* ^2^ > 2σ(*F* ^2^)] = 0.024
*wR*(*F* ^2^) = 0.064
*S* = 1.03
1763 reflections
118 parameters
H-atom parameters constrained
Δρ_max_ = 0.25 e Å^−3^
Δρ_min_ = −0.21 e Å^−3^

### 

Data collection: *COLLECT* (Nonius, 1998 ▶); cell refinement: *COLLECT*; data reduction: *DENZO* and *SCALEPACK* (Otwinowski & Minor, 1997 ▶); program(s) used to solve structure: *SIR97* (Altomare *et al.*, 1999 ▶) within *WinGX* (Farrugia, 2012 ▶); program(s) used to refine structure: *SHELXL97* (Sheldrick, 2008 ▶); molecular graphics: *ORTEP-3 for Windows* (Farrugia, 2012 ▶); software used to prepare material for publication: *SHELXL97* and *publCIF* (Westrip, 2010 ▶).

## Supplementary Material

Crystal structure: contains datablock(s) I, New_Global_Publ_Block. DOI: 10.1107/S1600536814002207/lh5658sup1.cif

Structure factors: contains datablock(s) I. DOI: 10.1107/S1600536814002207/lh5658Isup2.hkl

Supporting information file. DOI: 10.1107/S1600536814002207/lh5658Isup3.cml

CCDC reference:

Additional supporting information:  crystallographic information; 3D view; checkCIF report

## Figures and Tables

**Fig. 1.**
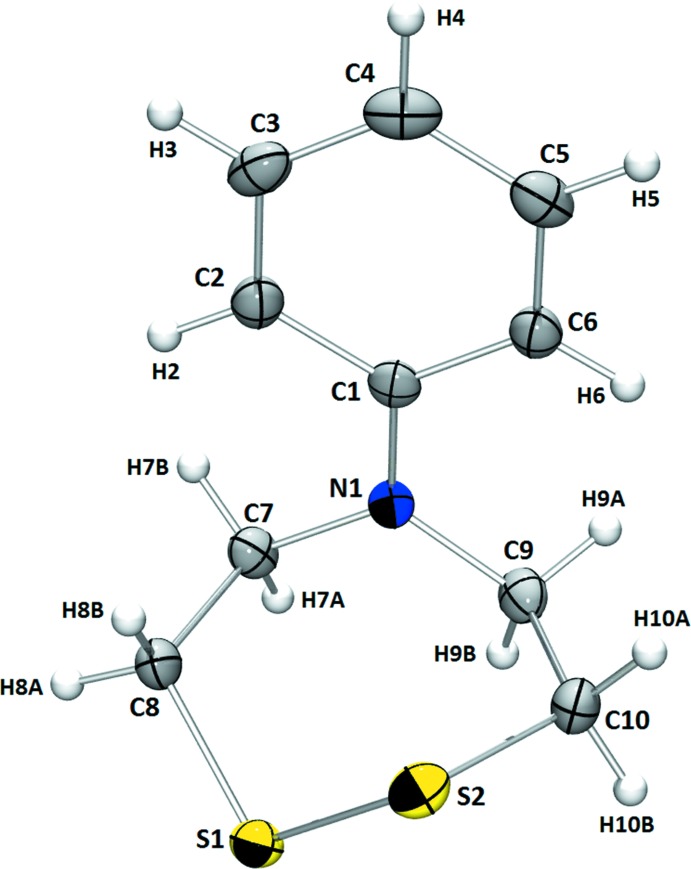
Molecular structure of title compound. Ellipsoids are drawn at the 50% probability level.

**Fig. 2.**
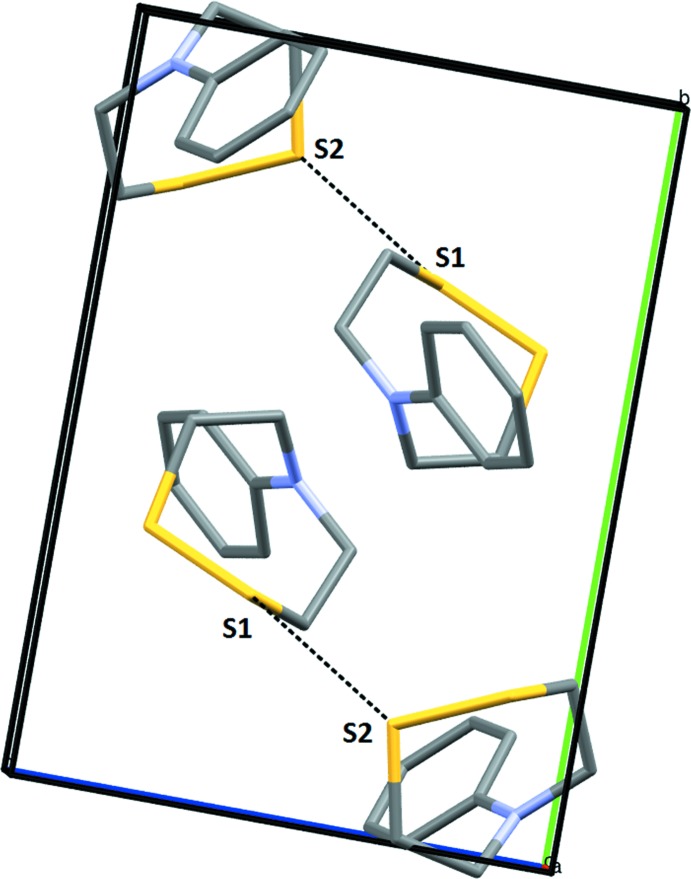
Part of the crystal structure viewed along the a axis. Interactions are shown between S1 and S2 of molecules related by the crystallographic c-glide.

## supplementary crystallographic information

### 1. Comment

Cyclic disulfides are of special interest because they are often a key component in many biologically relevant peptides (Pazderlová *et al.*, 2012). Because of this disulfide compounds are regularly found to have pharmacological activities. Herein is described the crystallographic properties of a 7-membered cyclic disulfide compound. The molecular structure of the title compound can be seen in Fig. 1.

The S—S bond distance is 2.0406 (5) Å. This value is comparable to other disulfide compounds. The mean average bond length for C—S—S—C bonds from 99 samples, reported by (Allen *et al.*, 1987) is 2.048 Å. The torsion in the C—S—S—C bonds is -83.89 (7)°, this compares similarly to the C—S—S—C torsion in the 7-membered ring disulfide 1,2,4,6-tetrathiacycloheptane reported in (Pickardt *et al.*, 2006), which has a C—S—S—C torsion of -89.4 (2)°.

The seven-membered ring adopts a chair conformation, as it does in the cyclic disulfide compound reported by Pickardt *et al.* (2006). The dominant intermolecular interactions are between S1···S2 of symmetry-related molecules. The contacts have a distance of 3.5246 (5) Å, this compares similarly to S···S interactions observed perviously by Chen *et al.* (2009) and Reinheimer *et al.* (2009) which are 3.396 (1) - 3.470 (1) Å and 3.580 (4) Å respectively.

The pyramidality of the amine functionality, measured by χn, the angle between the C1—N1 vector and the N1/C7/C9 plane, described by Allen *et al.* (1995), is 7.26 (15)°, indicating that the hybridization of the nitrogen atoms is mainly *sp^2^* (*sp^2^*χ_n_≈ 0°, *sp^3^*χ_n_≈ 60°).

### 2. Experimental

The title compound was prepared from *N*,*N*-bis(2-chloroethyl)aniline which had been prepared following literature methods reported by Elderfield *et al.* (1958). NaSH·H_2_O (1.08 g, 14.78 mmol) was stirred in ethanol (20 ml) under an argon atmosphere for 1 hr. *N*,*N*-bis(2-chloroethyl)aniline (0.5124 g, 2.35 mmol) was dissolved in ethanol (10 ml) under argon and then transferred into the NaSH·H_2_O solution *via* cannula. The reaction mixture was then heated to reflux for 24 hrs. The solvent volume was reduced by half *in vacuo* before degassed CH_2_Cl_2_ and H_2_O were added to the reaction flask and the product was extracted under argon. The organic phase was then transferred *via* cannula into a flask containing MgSO_4_. The product was isolated by filtration and removal of the solvent under vacuum. The X-ray quality crystals were obtained from a saturated dichloromethane solution of the title compound upon standing at 263 K for several days. Yield = 83%. ^1^H NMR (300 MHz, CDCl_3_): δ 7.25 (m, 2H), 6.85 (m, 3H), 3.52 (m, 4H), 2.74 (m, 4H).

### 3. Refinement

All H atoms were positioned geometrically and refined using a riding model, with C—H = 0.93–0.97 Å and with *U*_iso_(H) = 1.2*U*_eq_(C).

### Crystal data

**Table:**

C_10_H_13_NS_2_	*F*(000) = 448
*M**_r_* = 211.33	*D*_x_ = 1.392 Mg m^−^^3^
Monoclinic, *P*2_1_/*c*	Mo *K*α radiation, λ = 0.71070 Å
Hall symbol: -P 2ybc	Cell parameters from 1974 reflections
*a* = 9.5760 (2) Å	θ = 1.0–27.5°
*b* = 12.2310 (3) Å	µ = 0.48 mm^−^^1^
*c* = 9.9811 (2) Å	*T* = 153 K
β = 120.392 (2)°	Block, white
*V* = 1008.38 (4) Å^3^	0.50 × 0.30 × 0.20 mm
*Z* = 4	

### Data collection

**Table:**

Nonius Kappa CCD diffractometer	1763 independent reflections
Radiation source: fine-focus sealed tube	1675 reflections with *I* > 2σ(*I*)
Graphite monochromator	*R*_int_ = 0.012
ω scans	θ_max_ = 25.0°, θ_min_ = 4.1°
Absorption correction: multi-scan (*DENZO* and *SCALEPACK*; Otwinowski & Minor, 1997)	*h* = −11→11
*T*_min_ = 0.856, *T*_max_ = 1	*k* = −12→14
3055 measured reflections	*l* = −11→11

### Refinement

**Table:**

Refinement on *F*^2^	Primary atom site location: structure-invariant direct methods
Least-squares matrix: full	Secondary atom site location: difference Fourier map
*R*[*F*^2^ > 2σ(*F*^2^)] = 0.024	Hydrogen site location: inferred from neighbouring sites
*wR*(*F*^2^) = 0.064	H-atom parameters constrained
*S* = 1.03	*w* = 1/[σ^2^(*F*_o_^2^) + (0.0266*P*)^2^ + 0.5691*P*] where *P* = (*F*_o_^2^ + 2*F*_c_^2^)/3
1763 reflections	(Δ/σ)_max_ = 0.002
118 parameters	Δρ_max_ = 0.25 e Å^−^^3^
0 restraints	Δρ_min_ = −0.21 e Å^−^^3^

### Special details

**Table:**

Geometry. All e.s.d.'s (except the e.s.d. in the dihedral angle between two l.s. planes) are estimated using the full covariance matrix. The cell e.s.d.'s are taken into account individually in the estimation of e.s.d.'s in distances, angles and torsion angles; correlations between e.s.d.'s in cell parameters are only used when they are defined by crystal symmetry. An approximate (isotropic) treatment of cell e.s.d.'s is used for estimating e.s.d.'s involving l.s. planes.
Refinement. Refinement of *F^2^* against ALL reflections. The weighted *R*-factor *wR* and goodness of fit *S* are based on *F^2^*, conventional *R*-factors *R* are based on *F*, with *F* set to zero for negative *F^2^*. The threshold expression of *F^2^* > 2Σ(F^2^) is used only for calculating *R*-factors (gt)etc.and is not relevant to the choice of reflections for refinement. *R*-factors based on *F^2^* are statistically about twice as large as those based on *F*, and *R*-factors based on ALL data will be even larger.

### Fractional atomic coordinates and isotropic or equivalent isotropic displacement parameters (Å^2^)

**Table:**

	*x*	*y*	*z*	*U*_iso_*/*U*_eq_	
S1	1.04272 (4)	0.28440 (3)	0.62971 (4)	0.02268 (12)	
S2	1.07376 (4)	0.34787 (3)	0.83210 (4)	0.02409 (12)	
N1	0.74239 (13)	0.43703 (9)	0.57782 (13)	0.0190 (3)	
C1	0.65295 (16)	0.42454 (11)	0.65031 (15)	0.0187 (3)	
C2	0.66903 (17)	0.49796 (12)	0.76593 (16)	0.0218 (3)	
H2	0.7434	0.5548	0.7965	0.026*	
C3	0.57560 (17)	0.48654 (13)	0.83458 (16)	0.0261 (3)	
H3	0.5882	0.5360	0.9107	0.031*	
C4	0.46349 (18)	0.40283 (13)	0.79229 (17)	0.0282 (3)	
H4	0.4006	0.3960	0.8386	0.034*	
C5	0.44731 (17)	0.32959 (13)	0.67931 (17)	0.0261 (3)	
H5	0.3723	0.2732	0.6495	0.031*	
C6	0.54029 (17)	0.33875 (12)	0.61020 (16)	0.0223 (3)	
H6	0.5285	0.2877	0.5362	0.027*	
C7	0.72559 (17)	0.36111 (11)	0.45867 (16)	0.0217 (3)	
H7A	0.7562	0.3982	0.3915	0.026*	
H7B	0.6125	0.3407	0.3958	0.026*	
C8	0.82641 (17)	0.25754 (11)	0.52070 (17)	0.0224 (3)	
H8A	0.8026	0.2096	0.4342	0.027*	
H8B	0.7956	0.2197	0.5873	0.027*	
C9	0.87194 (17)	0.51649 (11)	0.63274 (17)	0.0231 (3)	
H9A	0.8296	0.5868	0.6406	0.028*	
H9B	0.9037	0.5236	0.5550	0.028*	
C10	1.02434 (18)	0.49172 (12)	0.78971 (17)	0.0259 (3)	
H10A	1.0103	0.5229	0.8715	0.031*	
H10B	1.1157	0.5283	0.7926	0.031*	

### Atomic displacement parameters (Å^2^)

**Table:**

	*U*^11^	*U*^22^	*U*^33^	*U*^12^	*U*^13^	*U*^23^
S1	0.0207 (2)	0.0222 (2)	0.0268 (2)	0.00209 (13)	0.01317 (16)	0.00058 (13)
S2	0.0215 (2)	0.0287 (2)	0.0192 (2)	−0.00065 (14)	0.00822 (15)	0.00285 (13)
N1	0.0195 (6)	0.0174 (6)	0.0198 (6)	−0.0004 (4)	0.0097 (5)	−0.0012 (4)
C1	0.0171 (7)	0.0180 (7)	0.0177 (6)	0.0048 (5)	0.0062 (5)	0.0045 (5)
C2	0.0202 (7)	0.0199 (7)	0.0222 (7)	0.0019 (5)	0.0085 (6)	0.0003 (5)
C3	0.0253 (7)	0.0307 (8)	0.0217 (7)	0.0072 (6)	0.0115 (6)	0.0004 (6)
C4	0.0225 (7)	0.0376 (8)	0.0274 (7)	0.0064 (6)	0.0148 (6)	0.0083 (7)
C5	0.0178 (7)	0.0270 (8)	0.0296 (8)	0.0004 (6)	0.0090 (6)	0.0063 (6)
C6	0.0196 (7)	0.0209 (7)	0.0219 (7)	0.0010 (5)	0.0072 (6)	0.0000 (5)
C7	0.0223 (7)	0.0235 (7)	0.0180 (7)	0.0018 (6)	0.0091 (6)	−0.0007 (5)
C8	0.0215 (7)	0.0206 (7)	0.0242 (7)	−0.0005 (6)	0.0109 (6)	−0.0044 (6)
C9	0.0280 (7)	0.0183 (7)	0.0275 (7)	−0.0024 (6)	0.0173 (6)	0.0002 (6)
C10	0.0241 (7)	0.0238 (7)	0.0291 (8)	−0.0048 (6)	0.0130 (6)	−0.0069 (6)

### Geometric parameters (Å, º)

**Table:**

S1—C8	1.8171 (14)	C5—C6	1.379 (2)
S1—S2	2.0406 (5)	C5—H5	0.9300
S2—C10	1.8155 (15)	C6—H6	0.9300
N1—C1	1.3811 (18)	C7—C8	1.5216 (19)
N1—C7	1.4523 (18)	C7—H7A	0.9700
N1—C9	1.4478 (18)	C7—H7B	0.9700
C1—C2	1.408 (2)	C8—H8A	0.9700
C1—C6	1.410 (2)	C8—H8B	0.9700
C2—C3	1.382 (2)	C9—C10	1.537 (2)
C2—H2	0.9300	C9—H9A	0.9700
C3—C4	1.386 (2)	C9—H9B	0.9700
C3—H3	0.9300	C10—H10A	0.9700
C4—C5	1.386 (2)	C10—H10B	0.9700
C4—H4	0.9300		
			
C8—S1—S2	102.27 (5)	N1—C7—H7A	108.6
C10—S2—S1	104.34 (5)	C8—C7—H7A	108.6
C1—N1—C7	121.14 (11)	N1—C7—H7B	108.6
C1—N1—C9	121.04 (11)	C8—C7—H7B	108.6
C7—N1—C9	117.26 (11)	H7A—C7—H7B	107.6
N1—C1—C2	121.28 (12)	C7—C8—S1	112.92 (10)
N1—C1—C6	121.45 (12)	C7—C8—H8A	109.0
C2—C1—C6	117.26 (13)	S1—C8—H8A	109.0
C3—C2—C1	120.81 (14)	C7—C8—H8B	109.0
C3—C2—H2	119.6	S1—C8—H8B	109.0
C1—C2—H2	119.6	H8A—C8—H8B	107.8
C2—C3—C4	121.32 (14)	N1—C9—C10	116.32 (11)
C2—C3—H3	119.3	N1—C9—H9A	108.2
C4—C3—H3	119.3	C10—C9—H9A	108.2
C5—C4—C3	118.41 (14)	N1—C9—H9B	108.2
C5—C4—H4	120.8	C10—C9—H9B	108.2
C3—C4—H4	120.8	H9A—C9—H9B	107.4
C6—C5—C4	121.30 (14)	C9—C10—S2	115.45 (10)
C6—C5—H5	119.3	C9—C10—H10A	108.4
C4—C5—H5	119.3	S2—C10—H10A	108.4
C5—C6—C1	120.88 (14)	C9—C10—H10B	108.4
C5—C6—H6	119.6	S2—C10—H10B	108.4
C1—C6—H6	119.6	H10A—C10—H10B	107.5
N1—C7—C8	114.49 (11)		
			
C8—S1—S2—C10	−83.89 (7)	N1—C1—C6—C5	−177.53 (12)
C7—N1—C1—C2	179.96 (12)	C2—C1—C6—C5	1.6 (2)
C9—N1—C1—C2	8.78 (19)	C1—N1—C7—C8	−83.69 (15)
C7—N1—C1—C6	−0.92 (19)	C9—N1—C7—C8	87.82 (15)
C9—N1—C1—C6	−172.10 (12)	N1—C7—C8—S1	−62.44 (14)
N1—C1—C2—C3	178.13 (12)	S2—S1—C8—C7	73.74 (10)
C6—C1—C2—C3	−1.0 (2)	C1—N1—C9—C10	69.91 (16)
C1—C2—C3—C4	0.0 (2)	C7—N1—C9—C10	−101.60 (14)
C2—C3—C4—C5	0.4 (2)	N1—C9—C10—S2	32.78 (16)
C3—C4—C5—C6	0.2 (2)	S1—S2—C10—C9	44.11 (12)
C4—C5—C6—C1	−1.3 (2)		
